# Phytoplankton Supplementation Lowers Muscle Damage and Sustains Performance across Repeated Exercise Bouts in Humans and Improves Antioxidant Capacity in a Mechanistic Animal

**DOI:** 10.3390/nu12071990

**Published:** 2020-07-04

**Authors:** Matthew Sharp, Kazim Sahin, Matthew Stefan, Cemal Orhan, Raad Gheith, Dallen Reber, Nurhan Sahin, Mehmet Tuzcu, Ryan Lowery, Shane Durkee, Jacob Wilson

**Affiliations:** 1 The Applied Science & Performance Institute, Research Division, Tampa, FL 33607, USA; mstefan@theaspi.com (M.S.); rgheith@theaspi.com (R.G.); dreber@theaspi.com (D.R.); rlowery@theaspi.com (R.L.); jwilson@theaspi.com (J.W.); 2 Animal Nutrition Department, School of Veterinary Medicine, Firat University, Elazig 23200, Turkey; nsahinkm@yahoo.com (K.S.); corhan@firat.edu.tr (C.O.); nsahin@firat.edu.tr (N.S.); mtuzcu@firat.edu.tr (M.T.); 3 Lonza Consumer Health Inc., Morristown, NJ 07960, USA; shane.durkee@lonza.com

**Keywords:** phytoplankton, antioxidants, muscle damage, muscle recovery, muscle soreness

## Abstract

The purpose of this study was to investigate the impact of antioxidant-rich marine phytoplankton supplementation (Oceanix, OCX) on performance and muscle damage following a cross-training event in endurance-trained subjects. Additionally, an animal model was carried out to assess the effects of varying dosages of OCX, with exercise, on intramuscular antioxidant capacity. Methods: In the human trial, endurance-trained subjects (average running distance = 29.5 ± 2.6 miles × week^−1^) were randomly divided into placebo (PLA) and OCX (25 mg) conditions for 14 days. The subjects were pre-tested on a one-mile uphill run, maximal isometric strength, countermovement jump (CMJ) and squat jump (SJ) power, and for muscle damage (creatine kinase (CK)). On Day 12, the subjects underwent a strenuous cross-training event. Measures were reassessed on Day 13 and 14 (24 h and 48 h Post event). In the animal model, Wistar rats were divided into four groups (*n* = 7): (i) Control (no exercise and placebo (CON)), (ii) Exercise (E), (iii) Exercise + OCX 1 (Oceanix, 2.55 mg/kg/day, (iv) Exercise + OCX 2 (5.1 mg/kg/day). The rats performed treadmill exercise five days a week for 6 weeks. Intramuscular antioxidant capacity (superoxide dismutase (SOD), catalase (CAT), glutathione peroxidase (GSH-Px)) and muscle damage (CK and myoglobin (MYOB) were collected. The data were analyzed using repeated measures ANOVA and *t*-test for select variables. The alpha value was set at *p* < 0.05. Results: For the human trial, SJ power lowered in PLA relative to OCX at 24 h Post (−15%, *p* < 0.05). Decrements in isometric strength from Pre to 48 h Post were greater in the PLA group (−12%, *p* < 0.05) than in the OCX. Serum CK levels were greater in the PLA compared to the OCX (+14%, *p* < 0.05). For the animal trial, the intramuscular antioxidant capacity was increased in a general dose-dependent manner (E + Oc2 > E + Oc1 > E > CON). Additionally, CK and MYOB were lower in supplemented compared to E alone. Conclusions: Phytoplankton supplementation (Oceanix) sustains performance and lowers muscle damage across repeated exercise bouts. The ingredient appears to operate through an elevating oxidative capacity in skeletal muscle.

## 1. Introduction

Sport performance depends on the ability of an athlete to produce and sustain high levels of physical, technical, decision-making, and psychological skills throughout competition. The deterioration of any of these skills could appear as a symptom of fatigue. The phenomenon of fatigue is complex, with the underlying processes developing as exercise proceeds to ultimately manifest as a decline of performance. The issue of fatigue is compounded when there is an imbalance between recovery and exertion. Recent research has demonstrated that endurance and cross-training athletes partake in multiple high-intensity competition events, which limits the recovery opportunities. The result is extreme stress to the body which is not counterbalanced by proper rest [1]. The lack of rest between intra-competition bouts impairs regeneration and leads to decrements in strength, power, and endurance-based performance [2,3]. Previous research has demonstrated that functional impairments are strongly associated with an increase in oxidative stress [4,5], defined as disturbances in the homeostatic balance where oxidant capacity exceeds the antioxidant capacity, causing the redox state to be more pro-oxidizing [6]. Mechanistically, extreme exercise can elevate oxygen consumption by up to 20-fold over resting levels [7]. The heightened use of oxygen may result in a leakage of reactive oxygen species (ROS) such as superoxide dismutase (SOD). If not properly regulated, these changes can alter cellular structure and function, leading to performance declines [4,5].

While athletes find it challenging to change the demands of competition, they may improve their recovery in response to exercise through the consumption of nutraceuticals that are rich in antioxidants. In fact, it is commonplace for athletes to supplement with antioxidants under the conviction that they will enhance the recovery of muscle function and performance [8,9]. As a result, a fair amount of attention has been given to antioxidant supplements for exercise recovery, which is largely due to their capacity to enhance the endogenous support in diminishing oxidative damage by discarding ROS [10,11]. The majority of studies investigating the impact of antioxidant supplementation on exercise report that antioxidants can reduce oxidative stress [12]; however, the physiological implications of this effect are not well known in more practical competition environments. Furthermore, strong evidence supporting antioxidant supplementation as a protectant against muscle damage is incomplete, as most investigations do not consider oxidative stress makers and intramuscular enzymes in conjunction with the functional indices of muscle damage (e.g., losses in force and power) [12,13].

Dieticians have made a push for natural and sustainable super foods, which contain an array of performance aids [14]. In response, scientists have isolated a unique source of marine phytoplankton, microalga *Tetraselmis chuii* (Oceanix (OCX), Lonza Consumer Health, Morristown, NJ, USA). This microalga contains highly active antioxidant enzymes, particularly superoxide dismutase (SOD), which speeds the reaction that converts superoxide into ordinary molecular oxygen, thereby protecting cells from oxidative damage [15]. The ingredient was also found to upregulate glutathione peroxidase and catalase enzymes in human skeletal muscle myoblasts in vitro [15]. When isolated, antioxidants have been shown to aid in recovery [16,17,18,19]. However, the effects on recovery of a marine-derived, SOD-rich ingredient remains to be investigated on high-intensity, cross-training events. Therefore, the purpose of this study was to investigate the targeted marine phytoplankton supplementation on recovery across repeated bouts of activity encompassing endurance, strength, and power and to investigate its effects on intramuscular antioxidant capacity using human and animal models. We hypothesized that marine phytoplankton would better reduce muscle damage while sustaining endurance and strength, which collectively indicates improved recovery. We also hypothesized that the ingredient would improve antioxidant capacity and lower oxidative stress in our mechanistic animal model.

## 2. Materials and Methods

### 2.1. Human Modal

#### 2.1.1. Subjects

Subjects were recruited by word of mouth, email contact, and direct contact with the local runner’s clubs. Subjects were excluded from the study if they: had a body mass index (BMI) ≥30 kg/m^2^, had allergies to fish, shellfish, algae, or seaweed; had any cardiovascular, metabolic, or endocrine disease; had undergone surgery that affects digestion and absorption, smoke, drink heavily (>7 and >14 drinks per week for women and men, respectively), were pregnant or planning to be pregnant, were on medication to regulate blood glucose, lipids, and/or blood pressure; had used anabolic–androgenic steroids, were currently using antioxidant supplements, non-steroidal anti-inflammatory drugs, or nutritional supplements known to stimulate recovery or muscle mass gains. A total of 20 subjects volunteered for participation in the study. Eighteen of the enrolled subjects completed the study and two subjects withdrew from the study due to work or family requirements. Prior to engaging in any study procedures, the subjects signed a written informed consent form that was approved by an Institutional Review Board (IntegReview, Austin, TX; Protocol # 1219) for study participation. Subjects in the study were considered active athletes who could withstand multiple endurance bouts (i.e., at least ran 20 miles × week^−1^). The self-reported weekly running mileage ranged from 20–60 miles (mean ± SD = 29.5 ± 10.9 miles × week^−1^). The subject characteristics are reported in Table 1 as the mean ± standard error.

#### 2.1.2. Study Protocol

This study was carried out in a randomized, double-blind, placebo-controlled and parallel manner. Prior to allocation into conditions, the subjects were assessed for maximal oxygen uptake (VO_2_max) on a graded treadmill test. The subjects were then classified into quartiles according to the VO_2_max values, and then subjects forming each quartile were randomly assigned to conditions. Following the condition allocation, the subjects underwent baseline testing on day 0 (Pre) which included: salivary cortisol, serum creatine kinase, maximal isometric strength, maximal muscle power, one-mile timed run, and perceptual measures using visual analog scales (VAS). Immediately following pre-testing, the subjects were given their respective condition (Oceanix (OCX) or microcrystalline cellulose-based placebo (PLA)). The OCX ingredient was independently examined by Brunswick Laboratories (Southborough, MA, USA) for oxygen radical absorbance capacity (ORAC) expressed in micromole Trolox equivalency (µmole TE) per gram. The results indicated that the values were high for hydroxyl radicals at 178.71 µmole TE/gram and super oxide anions at 348.11 µmole TE/gram, moderate in peroxynitrite and peroxyl radicals at 8.65 and 29.65 µmole TE/gram, respectively, and not detectable in singlet oxygen and hypochlorite. The ORAC values for the super oxide anion corresponded with high total values (38,000 IU per 100 g) of SOD in the raw powder measured.

The conditions were stored in visually identical capsules and containers. The subjects were required to consume one serving (25 mg) a day, either 30 min prior to exercise or with the first meal of the day on non-exercise days. Supplement compliance was assessed by supplement logs and the collection of supplement containers. The subjects were instructed to refrain from consuming any nutritional supplements for the duration of the study. Additionally, the subjects were informed to follow their habitual routine regarding exercise and diet for days 1–9 of the supplement period; however, the subjects were instructed to refrain from exercise on days 10 and 11 to avoid a potential interference effect from the cross-training bout on day 12. On the twelfth day of supplementation, the subjects returned to the laboratory to complete a supervised cross-training, muscle damage protocol. The subjects continued to supplement for two days after completing the supervised training protocol. Approximately 24 h and 48 h post training (days 13 and 14), the subjects were retested in a manner identical to the Pre to assess the changes in endurance, strength, power, and muscle damage. Study procedures are further described below.

#### 2.1.3. Maximal Oxygen Uptake (VO_2_max)

Subjects began the assessment at a velocity of 3 mph with a grade of 0%. The velocity was increased by 1 mph at the top of each min, and the rating of perceived exertion (RPE) was recorded using the Borg Scale [20]. Once an RPE of 12 was reached, the velocity increments ceased, and the grade was increased by 1% at the top of each min. This process continued until volitional fatigue was reached or if two of the three following criteria was reached: (1) leveling off (plateau) of oxygen uptake with an increase in work rate [21], (2) respiratory exchange ratio (VCO_2_/VO_2_) greater than 1.10 [22], or (3) 90% of the age-predicted maximum heart [23]. The Cardio Coach CO_2_ metabolic analyzer (Korr Medical Technologies Inc., Salt Lake City, UT, USA) was used for the collection and analysis of respiratory gases. This device has been validated for assessing maximal and submaximal VO_2_ [24]. The samples of respiratory gases were sampled every 15 s and measured using a 5 L mixing chamber technique. A Hans Rudolph one-way valve and silicone face mask was used for the gas collection. A 6-foot breathing tube connected the non-breathing valve to the mixing chamber inlet. Ventilatory oxygen was calculated using modified Haldane equations while the CO_2_ was measured directly by the analyzer.

#### 2.1.4. Salivary Cortisol (sCT)

Salivary cortisol samples were collected using IPRO Oral Fluid Collector (OFC) Kits (Soma Bioscience; Wallingford, UK). The OFC kits collect 0.5 mL of oral fluid and contain a color-changing volume adequacy indicator within the swab, giving collection times typically in the range of 20–50 s [25]. All samples were collected following a 10 h overnight fast. The samples were analyzed using an IPRO POC Lateral Flow Device (LFD), specific for cortisol, in an IPRO LFD Reader. Two drops of saliva/buffer mix from the OFC were added to the sample window of the LFD. The liquid runs the length of the test strip via lateral flow, creating a control and test line visible in the test window. Ten min after the sample was added, the test line intensity was measured in an IPRO LFD Reader and converted into a quantitative value.

#### 2.1.5. Serum Creatine Kinase (CK)

Venous blood was extracted by the venipuncture of the antecubital vein using a 21-gauge syringe and collected into a 10 mL ethylenediaminetetraacetic acid (EDTA) vacutainer tube (BD Vacutainer, Becton, Dickinson and Company, Franklin Lakes, NJ, USA) by a certified phlebotomist. Afterward, the blood samples were centrifuged at 2500 rpm for 10 min at 4 °C. The resulting serum were then aliquoted and stored at −80 °C until further analysis. The serum creatine kinase was assayed via commercially available ELISA kits. The samples were thawed once and analyzed in duplicate in the same assay for each analyte to avoid compounded inter-assay variance.

#### 2.1.6. Maximal Isometric Muscle Strength

Each subject was tested for maximal isometric strength using isometric mid-thigh pull (IMTP) performed in an Olympic style half rack to allow the fixation of the bar at any height. Subjects were secured to the bar using lifting straps and athletic tape. Utilizing a pronated clean grip, the subjects were instructed to assume a body position similar to the second pull of the snatch and clean. Knee angle was confirmed between 125° and 135° using a hand-held goniometer and the hip angle was approximately set at 175°. Once the body positioning was stabilized, the subject was given a countdown. Minimal pre-tension was allowed to eliminate slack prior to the initiation of the IMTP. Each subject performed two warm-up reps, one at 50% and one at 75% of the perceived maximum effort. Thereafter, subjects completed 2 maximal IMTPs separated by 2–3 min rest. Subjects were instructed to pull fast and hard and were given strong verbal encouragement during the assessment. Peak isometric force production was recorded using a linear position transducer [26].

#### 2.1.7. Maximal Muscle Power

Maximal muscle power was assessed via a countermovement jump (CMJ) and squat jump (SJ) on a dual force plate platform (Leonardo Mechanograph GRFP XL; Novotec Medical GmbH, Pforzheim, Germany). The platform was composed of two symmetrical force plates that separated the platform into a left and a right half. The resonance frequency of each plate was at 150 Hz. Each plate contained four strain gauge force sensors (the whole platform thus had eight force sensors). The sensors were connected to a laptop computer via a USB 2.0 connection. The signal from the force sensors was sampled at a frequency of 800 Hz and was analyzed using the Leonardo Mechanography GRFP Research Edition software (in this study version 4.2-b05.53-RES was used).

Prior to the test, the subjects completed a warm-up of 10 body weight squats and two submaximal effort CMJs. The subjects were instructed to stand in a comfortable and upright position with the feet about shoulder width apart and parallel to each other. The subjects then performed a countermovement by flexing the hips and knees. Once the subjects reached a preferred countermovement depth, they explosively extended their hip, knee, and ankle joints to perform a maximal vertical jump. Subjects performed 3 hands-free, maximal effort CMJs with 30 s rest between jumps. After completing 3 CMJs, the subjects rested for 2 min and performed 3 hands-free, maximal effort SJs separated by 30 s rest. The SJ started from a similar upright position as the CMJ and subjects were instructed to lower into a squat position (90° knee angle). The subjects held their squat position for approximately 3 s until a “jump” command was announced by the researcher. Immediately following the command, the subjects jumped as high as possible from the squat position without reloading or further descent.

#### 2.1.8. One-Mile Timed Run

As a warm-up, the subjects walked on a treadmill for 3 min at 4.8 km/h. Afterward, the subjects ran on the treadmill for 1 min at 9.7 km/h. Immediately following the warm-up, the subjects were allotted a 2 min period to rest or stretch. Thereafter, the treadmill grade and velocity settings were adjusted to a 1% incline and 4.8 km/h, respectively. The subjects maintained this velocity until the distance covered reached 0.05 miles. Upon reaching this distance, the subjects could control the velocity on the treadmill to complete one mile (marked as 1.05 miles) as fast as possible. Control panel placements displaying speed and time were covered to block the subject view. Time was kept by a research technician using a stopwatch. One-mile completion time was recorded to the nearest whole second.

#### 2.1.9. Perceptual Measures

The perceptual measures collected for the study were the perceived recovery status (PRS) scale, rating of perceived soreness (RPS), and the rating of perceived exertion (RPE). PRS was collected in a manner describe by Laurent et al. [27]. The PRS and RPS scales consist of a scalar representation numbering from 0–10. On the PRS scale, the visual descriptors of “very poorly recovered”, “adequately recovered” and “very well recovered” for perceived recovery are presented at numbers 0, 5, and 10, respectively. On the RPS scale, the visual descriptors of “no soreness at all”, “moderate soreness”, and “extreme soreness” are presented at numbers 0, 5, and 10, respectively. The subjects provided PRS at baseline (Pre), immediately before the training protocol (Pre2), and prior to the performance testing at 24 h and 48 h post-workout (24 h Post and 48 h Post) and the RPS was collected at Pre2, 24 h Post, and 48 h Post. The standard 6–20 Borg Scale was used to assess the RPE [20], and the RPE was collected four times: immediately after the 1 mile timed run at baseline (Pre), immediately after the training protocol, and immediately after the 1-mile timed run at 24 h Post and 48 h Post training protocol. Each perceptual measure was recorded as an arbitrary unit (au). 

#### 2.1.10. Cross-Training Muscle Damaging Event

Prior to the muscle-damaging session, the subjects completed a dynamic warm-up. Following the warm-up, the subjects completed a cross-training obstacle style program, which was anticipated to induce fatigue and muscle damage. The protocol consisted of 2 separate “blocks”. Each block contained 2 exercise stacks, with each stack containing 3 movements (e.g., A1, A2, and A3). The total amount of exercises performed during the training session was 12. Each exercise consisted of 10 reps per set, except for a sled push (45 meters push) and a rowing machine (300 m meters row). Each exercise stack was performed 3 times before moving on to the second stack of each block. Once Block 1 was completed, subjects moved to Block 2. Up to 2 min of rest was allowed between stacks, and then between blocks. However, it was at the discretion of the subject whether they used the full 2 min to rest. Minimal rest was given between exercises within a superset. The subjects were instructed to attempt to finish each stack in as little time as possible with compromising exercise form. An overview is provided in Table 2.

#### 2.1.11. Statistical Analysis

Prior to carrying out inferential statistics, normality was assessed via Shapiro–Wilk testing and the visual inspection of box blots. All data passed normality testing except for CK, which was logged transformed (y = log(y)) and reported as an arbitrary unit (au). Dependent variables were scrutinized using a two-way mixed analysis of variance (ANOVA) with the condition as the “between-group” factor, time as the “within-group” factor, and the subjects as a random factor. Whenever a significant *F*-value was obtained, post-hoc testing was performed with a Bonferroni correction for multiple comparisons. Additionally, the absolute mean differences (Time_2_–Time_1_) were analyzed using a two-tailed, unpaired *t*-test. The alpha level was set at *p* < 0.05 for statistical significance. For significant within- and between group differences, the mean difference (mean_diff_), 95% confidence interval (95% CI), and *p*-value were reported in text. The data are presented as the mean ± standard error.

### 2.2. Animal Modal

#### 2.2.1. Animals and Protocol

Male Wistar albino rats (*n* = 28, 8 weeks old) were provided from the Laboratory Animal Research Center, Firat University (Elazig, Turkey). The animals were kept in a room with standard conditions (22 ± 2 °C temperature, 55 ± 5% humidity, a 12 h light–12 h dark cycle). The ethical permission of the experiment was obtained from the Animal Experimentation Ethics Committee of Firat University (2019/139–206) according to the relevant laws, guidelines, and restrictions.

Rats were randomly divided into four groups (*n* = 7): (i) Control (no exercise and placebo), (ii) Exercise (E), (iii) Exercise + Oceanix 1 (2.55 mg/kg/day, (E + Oc1)), and (iv) Exercise + Oceanix 2 (5.1 mg/kg/day (E + Oc2)). Oceanix and placebo (physiological saline) were administered orally via gavage every day before exercise during the experiment period (6 weeks).

The rats were subjected to treadmill exercise on a motorized rodent treadmill (Commat Limited, Ankara, Turkey). The treadmill contained a stimulus grid at the back end of the treadmill giving an electric shock when the animal placed its paw on the grid. The apparatus had a 5-lane animal exerciser utilizing a single belt unit divided with walls suspended over the tread surface. In order to eliminate the diurnal variations, all the exercise tests were applied during the same time of the day. A week of adaptation was provided as pre-training practice for the animals in order to get familiar with the treadmill equipment and handling. In doing so, the rats in the exercise training groups were accustomed to treadmill exercise over a 5 day period such that: (i) first day, 10 m/min, 10 min, (ii) second day, 20 m/min, 10 min, (iii) third day, 25 m/min, 10 min, (iv) fourth day, 25 m/min, 20 min and (v) fifth day, 25 m/min, 30 min. Upon the adaptation of a week to the treadmill system for the novel and stress impacts, the rats in the treadmill exercise groups ran on the treadmill 25 m/min, 45 min/day and five days per week for 6 weeks according to the protocol described by Liu et al. [28]. The exercise model was chosen because it is the most common procedure to carry out animal exercise training from weeks to months at 45 min–1 h/day and 5 days/week. In addition, the exercise model provides adaptations to the cardiovascular system including the physiological remodeling of the heart representative with the increased O2 consumption, the improvement of cardiac contractile function, and calcium handling [29]. The chosen long-term animal exercise model fits an effective program to benefit both healthy and individuals at cardiovascular risk [30].

#### 2.2.2. Biochemical Analysis

Serum samples were obtained by taking blood samples to gel biochemical tubes after centrifugation (5000 rpm at 4 °C for 10 min). For the assays, the muscle tissue samples were homogenized within 10 min in 10 volumes of cold Tris 10 mM (pH 7.4). The serum concentrations of creatine kinase (CK) were assayed using a portable automated chemistry analyzer (Samsung LABGEO PT10, Samsung Electronics Co., Suwon, Korea). ELISA was also used in measuring the serum myoglobin (MyBioSource, San Diego, CA, USA). Tissue homogenates (10%, *w/v*) were prepared in 10 mM phosphate buffer and centrifuged at 13.000× *g* for 10 min at 4 °C. The muscle activities of superoxide dismutase (SOD), catalase (CAT) and glutathione peroxidase (GSH-Px) were determined using the commercially available kits (Cayman Chemical, Ann Arbor, MI, USA) according to the manufacturer’s procedure.

#### 2.2.3. Statistical Analysis

Data were presented as the mean ± standard error. All the tests were performed with the SPSS software program (IBM SPSS, Version 21.0; Chicago, IL, USA). Significance was determined by one-way ANOVA. Whenever a significant *F*-value was obtained, the Tukey HSD post-hoc test was applied for comparisons. Statistical significance for the data was defined as *p* < 0.05. 

## 3. Results

### 3.1. Human Trial

#### 3.1.1. Salivary Cortisol and Serum Creatine Kinase

**Figure 1:**
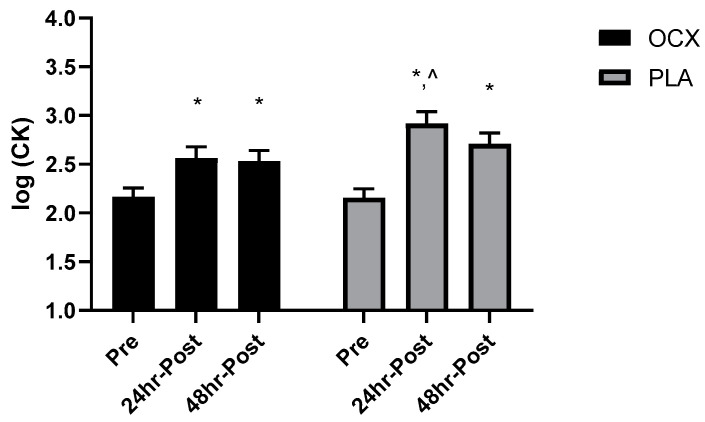
Bar charts displaying the mean and standard error for the log transformation of the plasma creatine kinase (CK) in the Oceanix (OCX) and placebo (PLA) conditions collected prior to the supplement period (Pre), one day, and two days following the supervised cross-training bout (24 h Post and 48 h Post, respectively). * = significantly greater than Pre (*p* < 0.05). ^ = significantly greater than OCX (*p* < 0.05).

There was no significant between-group difference for any dependent variable at Pre (*p* > 0.05). No significant differences were detected for sCT (*p* > 0.05). A significant group-by-time interaction was demonstrated for CK (*p* < 0.05). The post-hoc analysis indicated that both groups had higher CK levels at 24 h Post (OCX: mean_diff_ = 0.395 au, 95% CI: 0.153 to 0.637 au, *p* < 0.001; PLA: mean_diff_ = 0.759 au, 95% CI: 0.517 to 1.00 au, *p* < 0.0001) and 48 h Post (OCX: mean_diff_ = 0.364 au, 95% CI: 0.122 to 0.606 au, *p* < 0.005; PLA: mean_diff_ = 0.554 au, 95% CI: 0.312 to 0.796 au, *p* < 0.0001). However, 24 h Post levels were significantly higher in the PLA compared to the OCX (mean_diff_ = 0.354 au, 95% CI: 0.026 to 0.715 au, *p* < 0.05; Figure 1).

#### 3.1.2. Muscle Strength and Power

**Figure 2:**
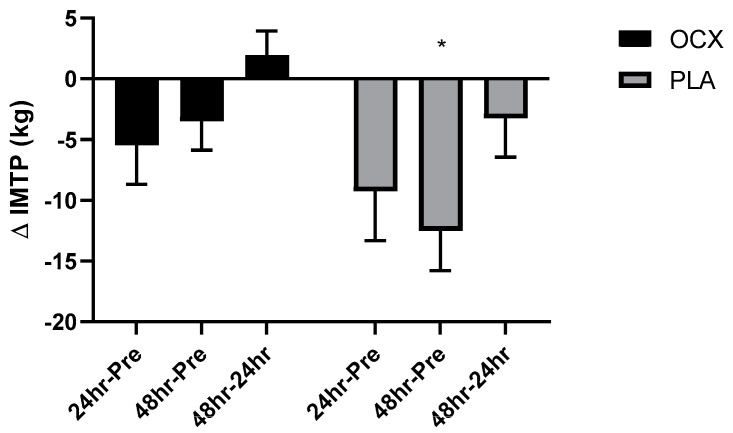
Bar charts displaying the mean and standard error for the absolute mean difference in maximal strength assessed by the isometric mid-thigh pull (IMTP) in the Oceanix (OCX) and placebo (PLA) conditions from Pre to 1 day following the cross-training bout (24 h Pre), Pre to 2 days following the cross-training bout (48 h Pre), and 1 day to 2 days following the cross-training bout (48 h–24 h). * = significantly lower than OCX (*p* < 0.05).

**Figure 3:**
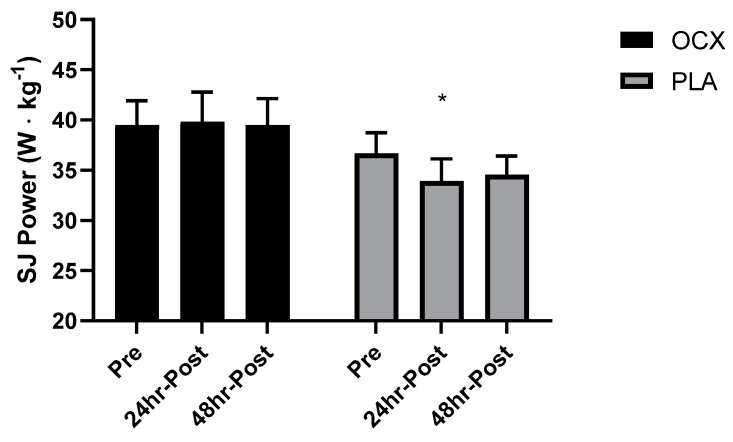
Bar charts displaying the mean and standard error for the relative power output during the squat jump (SJ) in the Oceanix (OCX) and placebo (PLA) conditions collected prior to the supplement period (Pre), one day, and two days following the supervised cross-training bout (24 h Post and 48 h Post, respectively). * = significantly lower than OCX (*p* < 0.05).

The absolute mean difference in IMTP strength from Pre to 48 h Post was significantly lower in the PLA group (mean_diff_ = −9.01 kg, 95% CI: −17.6 to −0.4 kg, *p* < 0.05; Figure 2). A significant main effect of time was detected for the CMJ relative power (*p* < 0.05) in which levels at 24 h Post (mean_diff_ = −1.63 W kg^−1^, 95% CI: 0.27 to 2.99 W kg^−1^, *p* < 0.05 and 48 h Post (mean_diff_ = −2.07 W kg^−1^, 95% CI: 0.71 to 3.43 W kg^−1^, *p* < 0.01) were significantly lower than Pre. A significant group-by-time interaction was demonstrated for the SJ relative power (*p* < 0.05). The post-hoc analysis revealed that the PLA was significantly lower than OCX at 24 h Post (mean_diff_ = −5.93 W kg^−1^, 95% CI: −14.32 to −0.31 W kg^−1^, *p* < 0.05; Figure 3).

#### 3.1.3. One Mile Timed Run

A significant main effect for time was detected for the one mile timed run (*p* < 0.0001) whereby times at 24 h Post (mean_diff_ = 79 s, 95% CI: 45 to 113 s, *p* < 0.0001) and 48 h post (mean_diff_ = 70 s, 95% CI: 36 to 104 s, *p* < 0.0001) were greater than Pre.

#### 3.1.4. Perceptual Measures

A significant main effect of time was detected for Soreness (*p* < 0.0001). The post-hoc analysis indicated that Soreness levels were elevated at 24 h Post (mean_diff_ = 2.3 au, 95% CI: 0.9 to 3.8 au, *p* < 0.01) and 48 h Post (mean_diff_ = 3.8 au, 95% CI: 2.4 to 5.3, *p* < 0.0001) compared to Pre. Additionally, the levels were elevated at 48 h Post compared to 24 h Post (mean_diff_ = 1.5 au, 95% CI: 0.1 to 3.0 au, *p* < 0.05). A significant main effect of time was detected for the PRS (*p* < 0.0001). The post-hoc analysis revealed that the PRS was lower at 24 h Post (mean_diff_ = −2.3 au, 95% CI: −3.9 to −0.7 au, *p* < 0.01) and 48 h Post (mean_diff_ = −3.5 au, 95% CI: −5.2 to −1.9 au, *p* < 0.0001) compared to Pre. Moreover, 48 h Post was significantly lower than Pre2 (mean_diff_ = −2.7 au, 95% CI: −4.3 to −1.0 au, *p* < 0.001). No significant differences were detected for RPE.

### 3.2. Animal Modal

#### 3.2.1. Muscle Damage

**Figure 4:**
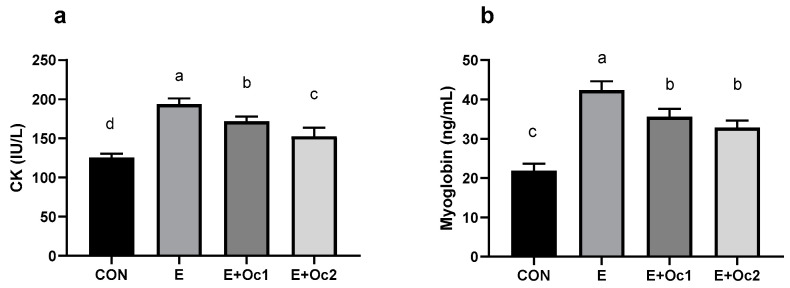
Bar charts displaying the mean and standard error for serum creatine kinase (**a**) and myoglobin (**b**) following the 6-week motorized rodent treadmill exercise protocol described in Section 3.1. Conditions are: Control (CON), Exercise (E), Exercise + Oceanix 1 (2.55 mg/kg/day, (E + Oc1)), and Exercise + Oceanix 2 (5.1 mg/kg/day (E + Oc2)). Conditions without a common letter differ significantly (*p* < 0.05).

For the exercise arm and both supplement arms, the CK was significantly greater than the control (Figure 4a). However, the CK in both supplement arms were lower than in the exercise arm, and E + Oc2 was lower than E + Oc1 (*p* < 0.05). The myoglobin concentration was significantly greater in the exercise and supplement arms compared to the control (Figure 4b). Additionally, both supplement arms were lower than the exercise arm (*p* < 0.05).

#### 3.2.2. Intramuscular Antioxidant Enzymes

Serum malonaldehyde (MDA) was lowered by exercise and exercise plus supplementation compared to control, and levels in E + Oc2 was significantly lower than E + Oc1 and E (*p* < 0.05). The exercise and supplement arms demonstrated higher SOD and GSH-Px relative to the control (*p* < 0.05). Furthermore, the supplement arms demonstrated higher levels compared to the exercise arm in a dose-dependent manner (i.e., E + Oc2 > E + Oc1 > E). Catalase was significantly greater in the exercise and supplement arms compared to the control, and both supplement arms were greater than the exercise arm (*p* < 0.05). The raw mean and standard error data for these biochemical measures are provided in Table 3.

## 4. Discussion

The purpose of this study was to investigate targeted marine phytoplankton supplementation (Oceanix, OCX) on recovery across repeated bouts of activity encompassing endurance, strength, and power, and to investigate its effects on intramuscular antioxidant capacity using human and animal models. We hypothesized that OCX would better reduce muscle damage and sustain endurance and strength, which collectively indicates improved recovery. We also hypothesized that the ingredient would improve the antioxidant capacity and lower oxidative stress in our mechanistic animal model. The primary findings of this study supported four out of our five major hypotheses. Specifically, OCX was able to lower muscle damage, sustain power and prevent declines in strength across repeated endurance and cross-training bouts. Mechanistically, the ingredient appears to operate through elevating oxidative capacity in skeletal muscle, which led to decreased oxidative stress and muscle damage.

Excessive exercise during competitions may contribute to disturbances in biological function through a physiological deregulation that leads to impairments in performance and recovery [31]. While athletes find it challenging to change the demands of a competition, they may alter their responses through the consumption of supplements that are rich in ingredients capable of influencing recovery. Previous investigations have taken an isolated approach to supplement research by determining their effects on recovery from either aerobic or anaerobic activities. However, many sports (e.g., soccer, basketball, and hockey) require individuals to be exposed to concurrent training stimuli [32]. Moreover, the emergence of CrossFit as a sport has driven athletes to excel across multiple physiological domains [33]. The uniqueness of the present study was that subjects participated in two endurance uphill runs, one cross-training event, and six maximal strength and power bouts (three maximal attempts on two occasions). We feel that this protocol allows researchers to truly determine the ecological validity of the present nutraceutical-based intervention.

Strength and power are two of the most critical attributes underlying success in competitions [32,34]. These variables are intimately related and allow athletes to be successful in their respective sport [35]. Moreover, the ability to sustain repeated outputs over distances is commonly referred to as endurance [32]. In the short term, these opposite spectrum attributes appear to compete with one another [32]. For example, just 25 min of endurance exercise ranging from 40 to 100% VO_2_ max acutely decreased power and strength by 19–36% [36]. From another perspective, resistance training acutely impairs endurance in a volume and intensity-dependent manner [37]. The present study found that repeated concurrent bouts of strength, power, and endurance decreased the majority of performance metrics examined in this study and resulted in noticeable muscle damage.

When observing CMJ power, it was found that performance dropped equally in both groups. Intriguingly, however, SJ power declined more in the PLA compared to the OCX group (−15% at 24 h Post). These differences were paralleled by greater increases in muscle damage in the PLA (+14% at 24 h Post) compared to the OCX. The ability of OCX to improve power in the SJ but not the CMJ may be due to the differences in mechanics between the two exercises [38]. Specifically, athletes may be able to better use passive elastic components of connective tissue and tendons to overcome muscle damage in a CMJ relative to a SJ, which removes much of the ability to store and release elastic energy [39]. Intriguingly, the ability to use elastic energy increases as the stretch-shorten cycle time decreases [39]. Correspondingly, the high intensity run in our study would demonstrate the shortest stretch shortening cycle in all the measures examined. Thus, the lack of differences in endurance between conditions despite greater muscle damage in the PLA may be explained by similar reasons to the CMJ. In support, we found that decrements in our isometric measure of strength from Pre to 48 h Post were greater in the PLA group (−12%) than OCX.

Since improving recovery through lowering muscle damage is of great interest to athletes, it is worth exploring how OCX may influence these outcomes. To begin to answer this question, it is important to understand the contribution of reactive oxygen species (ROS) to exercise-induced muscle damage. This topic has been addressed in impactful reviews [40]. Briefly, exercise elevates metabolism, and the use of oxygen is heightened [41]. The result is a leakage of ROS from the mitochondria [41]. Superoxide is a major ROS produced as a by-product of oxygen metabolism and, if not regulated, causes many types of cell damage [42]. As such, ROS alters cell structure and function, and contributes to muscle damage causing performance declines [42].

The ingredient administered in this study was derived from the microalga *Tetraselmis chuii*. This microalga contains highly active antioxidant enzymes, particularly superoxide dismutase (SOD), which speeds the reaction that converts superoxide into ordinary molecular oxygen, thereby protecting cells from oxidative damage [15]. The ingredient was also found to upregulate glutathione peroxidase and catalase enzymes in human skeletal muscle myoblasts in vitro [15]. Research has shown that antioxidant supplementation can protect against exercise-induced muscle damage [43,44] and as a result, reduce muscle performance loss and fatigue [45,46]. Our human and animal trials demonstrated that exercise increased muscle damage (e.g., elevated CK and myoglobin levels). Moreover, in both studies, muscle damage was lowered by supplementation. An independent analysis of the present ingredient demonstrated high antioxidant capacity via high concentrations of SOD (38,000 IU per 100 g) with robust corresponding ORAC values for the enzymes’ corresponding anion. These properties led us to explore multiple intramuscular antioxidant enzymes in exercise alone and exercise with OCX supplementation. We found that the ingredient improved measures of antioxidant capacity in a generally dose-dependent fashion.

The results of our study agreed with the past literature demonstrating that exercise alone increases antioxidant capacity, likely as an adaptation to increased oxidative stress [47]. Our animal study also found that exercise combined with the administration of the SOD rich ingredient further decreased oxidative stress. Our findings agreed with a number of antioxidant supplement studies, which demonstrated decreased oxidative stress. These studies include positive effects with vitamin E [48,49,50], vitamin C [10,51], polyphenolic compounds [52], β-carotene [53], and different antioxidant combinations [54]. These changes occurred as result of elevations in antioxidant capacity as indicated by greater levels of the three intramuscular enzymes measured. The increased capacity found agreed with the past research demonstrating that antioxidant supplementation can increase antioxidant enzyme activity both at rest [55] and in combination with exercise [51,54].

While the mechanism of action still needs exploring, previous in vitro research with the ingredient in human skeletal muscle myoblasts observed that the changes in the proteins studied in our research occurred via the transcriptional responses of genes encoding the antioxidant enzymes and the further regulation of the polypeptide translation downstream of these events [15]. Specifically, a parallel and positive response in enzyme activities and transcripts for SOD, CAT and GSH-Px encoding genes in myoblasts, as a consequence of microalga *Tetraselmis chuii* treatment, was found [15]. This previous finding appears to unravel the potential molecular basis of the cytoprotective effect of the studied ingredient in relation to the primary antioxidant enzymes investigated.

## 5. Conclusions

We can conclude that the training program was effective at inducing high levels of neuromuscular fatigue. However, marine phytoplankton supplementation (Oceanix, OCX) was able to improve recovery, sustain power, and prevent declines in strength across repeated endurance and cross-training bouts. Since recovery can be defined as, “returning what was lost due to exercise,” [56], we demonstrated that OCX supplementation can improve recovery from intense competitions. Moreover, we present for the first time mechanistic data, which support the OCX role in improving intramuscular antioxidant capacity when combined with exercise.

These findings may allow practitioners to better recommend supplementation for athletes competing under high stress scenarios. While no person should be expected to maximally display every skill of athleticism on a daily basis, OCX supplementation may assist in improving recovery during demanding training or competition periods.

## Figures and Tables

**Table 1:** Subject characteristics.

Variable	OCX (*n* = 9)	PLA (*n* = 9)
Age (years)	38 ± 1	36 ± 2
Height (cm)	171.3 ± 3.6	168.5 ± 2.8
Body Mass (kg)	73.0 ± 4.5	69.1 ± 3.3
BMI (kg/m^2^)	24.8 ± 1.0	24.4 ± 1.1
Body Fat (%)	27.2 ± 2.4	26.7 ± 2.4
VO_2_max (mL/kg/min)	46.0 ± 2.5	47.7 ± 2.9
OCX = Oceanix; PLA = Placebo; BMI = body mass index		

**Table 2:** Warm-up and cross-training protocol.

Warm-up	Block 1	Block 2
World’s Greatest Stretch 45 m	A1. Bulgarian Split Squat × 10e	A1. Goblet Squat × 10
Hamstring Scoops 45 m	A2. 45 m Sled Push	A2. 300 m Row
Lunge w/Overhead Reach 45 m	A3. Unilateral BW Calf Raise × 20e	A3. KB Swing × 10
High Knees 45 m		
Butt Kicks 45 m	B1. Med Ball Slam × 10	B1. DB Squat Thrusters × 10
Lateral Shuffle 45 m	B2. Inverted BW Row × 10	B2. 35 cm Drop Jump × 10
Shoulder Bilateral “T”, “W”, “Y” × 10	B3. 45 cm Step-up × 10e	B3. Reverse Lunge × 10e

m = meters; cm = centimeters; e = each leg; BW = bodyweight; DB = dumbbell; KB = kettlebell; W = prone shoulder internal/external rotation; T = prone horizontal shoulder abduction; Y = prone shoulder extension.

**Table 3:** Intramuscular activity of MDA, SOD, CAT, and GSH-Px across conditions.

Sample	Groups			
	Control	Exercise	E + Oc1	E + Oc2
MDA (µmol/L)	0.76 ± 0.02 ^a^	0.68 ± 0.01 ^b^	0.61 ± 0.03 ^b^	0.46 ± 0.02 ^c^
SOD (U/mL)	70.88 ± 1.57 ^d^	82.28 ± 1.82 ^c^	97.68 ± 2.36 ^b^	113.46 ± 1.26 ^a^
CAT (U/mL)	140.82 ± 2.80 ^c^	153.00 ± 2.85 ^b^	164.92 ± 2.12 ^a^	175.79 ± 3.91 ^a^
GSH-Px (U/mL)	71.13 ± 1.97 ^d^	82.00 ± 2.61 ^c^	95.29 ± 1.52 ^b^	107.10 ± 1.77 ^a^

Data are presented as mean and standard error a–d: Means in the same line without a common superscript differ significantly (*p* < 0.05). MDA = malonaldehyde; SOD = superoxide dismutase; CAT = catalase; GSH-Px = glutathione peroxidase.

## Abbreviations

OCX	Oceanix
MDA	Malonaldehyde
TE	Trolox equivalency
CK	Creatine kinase
MYOB	Myoglobin
IMTP	Isometric mid-thigh pull
CMJ	Countermovement jump
SJ	Squat jump
E	Exercise
E + Oc1	Exercise + Oceanix 1 (2.55 mg/kg/day)
E + Oc2	Exercise + Oceanix 2 (5.1 mg/kg/day)
SOD	Superoxide dismutase
CAT	Catalase
GSH-Px	Glutathione peroxidase
PLA	Placebo
sCT	Salivary cortisol
BMI	Body mass index
RPE	Rating of perceived exertion
PRS	Perceived recovery status
ANOVA	Analysis of variance
ROS	Reactive oxygen species
EDTA	Ethylenediaminetetraacetic acid
m	Meters
cm	Centimeters
e	Each leg
DB	Dumbbell
KB	Kettlebell
W	Prone shoulder internal/external rotation
T	Prone horizontal shoulder abduction
Y	Prone shoulder extension
