# Erratum: Sharp et al. Phytoplankton Supplementation Lowers Muscle Damage and Sustains Performance across Repeated Exercise Bouts in Humans and Improves Antioxidant Capacity in a Mechanistic Animal. *Nutrients* 2020, *12*, 1990

**DOI:** 10.3390/nu14030408

**Published:** 2022-01-18

**Authors:** Matthew Sharp, Kazim Sahin, Matthew Stefan, Cemal Orhan, Raad Gheith, Dallen Reber, Nurhan Sahin, Mehmet Tuzcu, Ryan Lowery, Shane Durkee, Jacob Wilson

**Affiliations:** 1 The Applied Science & Performance Institute, Research Division, Tampa, FL 33607, USA; mstefan@theaspi.com (M.S.); rgheith@theaspi.com (R.G.); dreber@theaspi.com (D.R.); rlowery@theaspi.com (R.L.); jwilson@theaspi.com (J.W.); 2 Animal Nutrition Department, School of Veterinary Medicine, Firat University, Elazig 23200, Turkey; nsahinkm@yahoo.com (K.S.); corhan@firat.edu.tr (C.O.); nsahin@firat.edu.tr (N.S.); mtuzcu@firat.edu.tr (M.T.); 3 Lonza Consumer Health Inc., Morristown, NJ 07960, USA; shane.durkee@lonza.com

## Modifications to the Main Text

The authors would like to correct a typographical error in their prior publication [1]. In the original article, there was a mistake regarding the treatment dosage in the animal model. The correct treatment dosage for Exercise + Oceanix 1 (E + Oc1) was 2.55 mg/kg/day, not 2.55 mg/day. Similarly, the correct dosage for Exercise + Oceanix 2 (E + Oc2) was 5.1 mg/kg/day, not 5.1 mg/day.

## Modifications to Back Matter Sections

**Author Contributions:** Conceptualization, M.S. (Matthew Sharp), K.S., R.L., S.D. and J.W.; data curation, M.S. (Matthew Sharp), M.S. (Matthew Stefan), C.O., R.G., D.R., N.S. and M.T.; formal analysis, M.S. (Matthew Sharp), C.O., N.S., M.T. and J.W.; investigation, M.S. (Matthew Sharp), M.S. (Matthew Stefan), C.O., R.G., D.R., N.S. and M.T.; methodology, M.S. (Matthew Sharp), K.S., M.S. (Matthew Stefan), C.O., R.G., D.R., N.S. and M.T.; supervision, M.S. (Matthew Sharp) and J.W.; Writing—Original draft, M.S. (Matthew Sharp), K.S. and J.W.; writing—review and editing, M.S. (Matthew Sharp), K.S., R.L. and J.W. All authors have read and agreed to the published version of the manuscript.

**Funding:** All projects reported in this manuscript was supported by Lonza Consumer Health Inc. (Morristown, NJ, USA).

**Institutional Review Board Statement:** The study was conducted according to the guidelines of the Declaration of Helsinki and approved by IntegReview IRB (protocol code: 1219, date of approval: 23 December 2019).

**Informed Consent Statement:** Informed consent was obtained from all subjects involved in the study.

**Data Availability Statement:** The data presented in this study are available on request from the corresponding author.

**Abbreviations**

E + Oc1 Exercise + Oceanix 1 (2.55 mg/kg/day)
E + Oc2 Exercise + Oceanix 2 (5.1 mg/kg/day)

The authors apologize for any inconvenience caused and state that the scientific conclusions are unaffected.
